# Awareness, Use, and Perceptions of Low-Carbohydrate Diets

**Published:** 2008-09-15

**Authors:** Lila J Finney Rutten, Amy Lazarus Yaroch, Uriyoán Colón-Ramos, Audie A Atienza

**Affiliations:** National Cancer Institute; National Cancer Institute, Bethesda, Maryland; National Cancer Institute, Bethesda, Maryland; National Cancer Institute, Bethesda, Maryland

## Abstract

**Introduction:**

Low-carbohydrate diets (LCDs) have regained popularity in recent years, but public awareness and perceived healthfulness of LCDs have not been explored. We describe population awareness, use, and perceptions of the healthfulness of LCDs and examine differences by sociodemographic and communication variables.

**Methods:**

Nationally representative data from the Health Information National Trends Survey (HINTS 2005) were analyzed by using multivariate logistic regression to examine independent correlates of awareness, use, and perceptions of the healthfulness of LCDs.

**Results:**

Awareness of LCDs in the United States was high (86.6%). Independent correlates of awareness included being a college graduate, being non-Hispanic white, and having a high body mass index (BMI). Among respondents who were aware of LCDs, approximately 17% had tried LCDs during the last year. Independent correlates of LCD use included being a woman and having a high BMI. One-third of respondents who were aware of LCDs agreed that they are a healthy way to lose weight. Independent correlates of perceived LCD healthfulness included not being a high school graduate and being likely to change behavior in response to new nutrition recommendations.

**Conclusion:**

This study is among the first to explore correlates of awareness, use, and perceptions of LCDs in a nationally representative sample. Despite high levels of awareness of LCDs, these diets are not used frequently and are not perceived as being healthy.

## Introduction

Since the publication of *Dr. Atkins' Diet Revolution* in 1972 (1), low-carbohydrate diets (LCDs) and high-protein diets have gained prominence in the United States. In recent years, LCDs have regained popularity, as evidenced by the publication of such books as *Dr. Atkins' New Diet Revolution *(2), *The New Sugar Busters!* (3), and *The South Beach Diet* (4).

In 2005, dietary experts from government, academia, and industry convened the International Life Sciences Institute North America Technical Committee on Carbohydrates to review scientific evidence about the healthfulness of LCDs. This committee identified gaps in existing research, including a need to assess awareness and trends in adoption of LCDs (5). Although efficacy (6) and use of LCDs have been explored (7), public awareness and perceived healthfulness of LCDs have not been examined in a nationally representative sample. Therefore, we examined public awareness, use, and perceptions of LCDs.

The purpose of our research was to use national data to explore the correlates of awareness, use, and perceptions of LCDs, including sociodemographic characteristics and key communication variables. The efficacy, effectiveness, and safety of LCDs have been a matter of scientific debate. Given the controversy surrounding these issues, we neither endorse nor denounce LCDs but rather describe national patterns. To our knowledge, the 2005 Health Information National Trends Survey (HINTS) provides the first available nationally representative data that document awareness, use, and perceptions of LCDs, and we help fill a gap in the literature by analyzing these data to reveal trends.

## Methods

### Data collected

We analyzed data from HINTS 2005. HINTS collects nationally representative data about the American public's need for, access to, and use of health-related information, including data that assess knowledge and attitudes about and behavior concerning nutrition and diet.

Data for HINTS were collected from February through August 2005. The list-assisted sample design followed a random-digit–dial format, in which all US telephone exchanges were included. One adult from each household was selected for an interview, which was conducted in English or Spanish on the basis of respondent preference. The total sample was 5,586 adults. The response rate for the household screener was 34.0%, and the response rate for extended interview was 61.3%, resulting in an overall response rate of 20.8%. All respondents provided sociodemographic information and answered questions about awareness, use, and perceptions about LCDs, and half of respondents were randomly assigned questions about nutrition-related behavior and information seeking. Details about sampling design are published elsewhere (8).

To assess behavioral reactions to nutrition recommendations, respondents were asked, "Think about the last time you heard a new recommendation about nutrition. Which of the following things did you do in response to the new recommendation?" Response options were coded dichotomously: "I changed what I do" and "I did not change what I do" or "I waited to get more information." To assess confusion about nutrition recommendations, respondents were asked to rate on a 4-point scale their agreement (*strongly agree* to *strongly disagree*) with the following statement: "There are so many different recommendations about nutrition that it's hard to know which ones to follow." Respondents estimated their level of exposure (*a lot*, *some*, *a little*, or *not at all*) to information about nutrition from 5 sources: 1) television, 2) newspapers, 3) magazines, 4) the Internet, and 5) health care professionals.

### Outcome variables

Outcome variables for our study were awareness of LCDs, use of LCDs, and perceptions about the healthfulness of LCDs. Responses to these 3 questions were yes or no. Awareness of LCDs was assessed for all respondents by asking a question about highly visible LCDs instead of providing an explicit definition of LCDs: "Are you aware of low-carbohydrate, high-protein diets such as the Atkins Diet, the Zone, Sugar Busters, or the South Beach Diet?" Use of LCDs was assessed by asking respondents who were aware of LCDs the following question: "Have you tried a low-carbohydrate, high-protein diet in the past 12 months?" (In our analyses, respondents who had never heard of LCDs (n = 584) were designated as having never tried LCDs.) Perception of the healthfulness of LCDs was assessed by asking respondents who were aware of LCDs the following question: "Do you think that a low-carbohydrate, high-protein diet is a healthy way to lose weight?"

### Data analyses

We used SUDAAN version 9.0.1 (RTI International, Research Triangle Park, North Carolina) to estimate standard errors of point estimates for the complex survey data. All data were weighted to provide representative estimates of the adult US population. Descriptive analyses were conducted for all variables. The Pearson correlation and the χ^2^ test were conducted to examine associations among variables. Multivariate logistic regression models were used to examine independent correlates of awareness, use, and perceived healthfulness of LCDs. Variables that were significantly (*P* <.05) associated with outcome variables in the bivariate analyses were included in the multivariate models to examine the unique variance contributed by each variable to the respective outcomes. For continuous variables included in the multivariate model, odds ratios (ORs) were calculated based on a 1-unit change in each measure.

## Results

Weighted percentages for sociodemographic characteristics of the sample are summarized in Table 1. Overall, awareness of LCDs was high; 86.6% of total respondents reported that they were aware of LCDs. Awareness was associated at the bivariate level with being a woman, being a college graduate, being non-Hispanic white, having an annual income of $50,000 or more, being 50 to 64 years of age, having an approximate mean body mass index (BMI) of 27 kg/m^2^, and reporting no behavioral change in response to new nutrition recommendations. Approximately 17% of respondents reported that they had tried an LCD during the past 12 months. Use of LCDs was significantly associated at the bivariate level with being a woman; being a college graduate; reporting a race or ethnicity other than non-Hispanic white, non-Hispanic black, or Hispanic; having an annual income of $50,000 or more; being aged 50 to 64 years; having an approximate mean BMI of 29 kg/m^2^; having been exposed to "a lot" or "some" nutrition information on the Internet during the past 12 months; and having been exposed to "a lot" or "some" nutrition information from a health care professional during the past 12 months. One-third (33.7%) of respondents perceived LCDs to be a healthy way to lose weight. Among respondents who reported that they had tried an LCD during the past 12 months, 58.1% reported that they thought it was a healthy way to lose weight (data not shown). Reported agreement with the healthfulness of LCDs was significantly associated at the bivariate level with being a man, having less than a high school education, being Hispanic, having an annual income <$25,000, reporting changing behavior in response to new nutrition recommendations, having been exposed to "a lot" or "some" nutrition information from a health care professional during the past 12 months, being ≥75 years of age, and having an approximate mean BMI of 27 kg/m^2^.

Table 2 displays the results of the multivariate analyses to examine independent correlates of awareness, use, and perceptions of LCDs. In model A, awareness of LCDs was higher among respondents with a high school education or more and among respondents with a high BMI. Awareness of LCDs was lower among non-Hispanic blacks and Hispanics than among non-Hispanic whites. In model B, use of LCDs was higher among women than among men and higher among respondents with a high BMI than among respondents with a low BMI. In model C, respondents with a high school or college degree were less likely to agree that LCDs are a healthy way to lose weight than were respondents with less than a high school education. Respondents who reported that they change their behavior in response to new nutrition recommendations were more likely to agree that LCDs are a healthy way to lose weight than were respondents who reported that they do not change their behavior.

## Discussion

Because of the lack of consensus among health professionals about LCDs, using these diets to manage weight is controversial. Insight into the correlates of awareness, use, and perceptions of LCDs helps show how sociodemographic characteristics and communication behaviors relate to the way people react to an environment of multiple and occasionally contradictory nutrition messages. Our results showed high awareness of LCDs among Americans, which is not surprising because data for HINTS 2005 were collected when LCDs were highly publicized in the media.

Respondents who were highly educated, were non-Hispanic white, and had a high BMI were most likely to be aware of LCDs. Among respondents who were aware of LCDs, those with a low level of education and who reported a high likelihood of changing their behavior in response to new nutrition recommendations were more likely to perceive LCDs as a healthy way to lose weight. This pattern of awareness is consistent with the "knowledge gap" theory that health knowledge is unequally distributed. This gap is characterized by a discrepancy between people from high socioeconomic status (SES) groups who tend to have more information (or are "information rich") than do people from low SES groups (who are "information poor") (9). Correlates of use and perceived healthfulness of LCDs were different from those of awareness, which suggests that factors that may influence use of LCDs do not necessarily drive perceptions of their healthfulness. More than half of respondents who had tried LCDs reported that they perceived them to be a healthy way to lose weight. Results also suggested that respondents who regard LCDs as a healthy way to lose weight may be more likely to pursue dietary recommendations before scientific evidence of efficacy and safety are available.

Estimates of LCD use in our sample were approximately 5 times greater than those found in a previous study, which reported a prevalence of 3.4% (7). However, the data used in that study were collected in 2002, and data used for our study were collected in 2005, which suggests that awareness and use of LCDs has increased over time.

### Limitations

HINTS 2005 was not a prospective study. Therefore, results of our analyses provide a cross-sectional view of public perceptions of LCDs. Response rates for HINTS 2005, although comparable to those of other national telephone surveys, reflect the low response rates for telephone surveys in general (10). The sample sizes for the multivariate analyses were restricted because models included items for which only subsamples were assessed. The multivariate results highlight the robust nature of the significant relationships identified. Many of the significant relationships identified at the bivariate level may not have emerged in the multivariate analyses because of lack of statistical power or collinearity with other variables. Additional research is warranted to clarify the significance of the variables that emerged in the bivariate analyses but not in the multivariate analyses. Data used in this study were self-reported and consequently have associated biases. Finally, no standardized questions assessing awareness, use, and perceptions of LCDs were available at the time of the data collection. However, questions that were developed for HINTS 2005 to capture new information about LCDs were carefully considered and revised through several rounds of cognitive interviews (8). Ideally, more variables of interest would have been included (eg, use of other diets, more detailed questions about LCDs), but because space on the survey was limited, we were able to analyze only the variables presented here.

### Conclusions

Results of our study provide insight into the sociodemographic and communication behavior correlates of awareness, use, and perceived healthfulness of LCDs in a nationally representative sample. This insight can shape efforts to promote awareness and use of evidence-based nutrition recommendations to bolster public knowledge of healthful dietary practices.

## Figures and Tables

**Table 1:** Awareness, Use, and Perceptions of Low-Carbohydrate Diets by Participants (N = 5,586)^a^ of the Health Information National Trends Survey (HINTS), by Sociodemographic, Behavioral, and Communication Variables, United States, 2005

**Characteristic**	No. Who Are Aware of LCDs (%)	**No. Who Used LCDs b** **(%)**	No. Who Perceive LCDs as Healthy (%)
**Total No. (%)**	4,844 (86.6)	1,015 (16.8)	1,408 (33.7)
**Sex**			
Male	1,591 (83.1)	291 (13.7)	503 (37.5)
Female	3,253 (89.8)	724 (19.6)	905 (30.5)
*P* value^c^	<.001	<.001	<.001
**Education level**			
<High school	444 (63.8)	104 (13.9)	194 (53.9)
High school graduate	2,719 (88.7)	543 (16.6)	803 (34.1)
College graduate	1,632 (96.0)	362 (19.7)	392 (24.2)
*P* value^c^	<.001	.02	<.001
**Race/ethnicity**			
Non-Hispanic white	3,848 (93.3)	787 (17.8)	1,012 (28.4)
Non-Hispanic black	348 (75.1)	68 (14.6)	132 (46.8)
Hispanic	329 (64.5)	78 (11.4)	149 (52.9)
Other	244 (81.8)	66 (21.4)	80 (40.6)
*P* value^c^	<.001	.009	<.001
**Annual household income (US $)**			
<25,000	953 (75.5)	174 (13.4)	351 (44.4)
25,000-49,999	1,094 (85.6)	224 (16.3)	313 (34.4)
50,000-74,999	880 (94.5)	206 (21.7)	238 (32.1)
≥75,000	1,110 (94.0)	272 (20.6)	291 (27.1)
*P* value^c^	<.001	.002	.001
**Change behavior in response to new nutrition recommendations^d^**			
Yes	344 (82.2)	101 (25.1)	145 (51.6)
No	1,232 (90.6)	294 (19.8)	372 (34.0)
*P* value^c^	.005	.17	.001
**Agree too many nutrition recommendations^d^**			
Agree	1,964 (86.7)	408 (16.3)	577 (32.9)
Disagree	442 (85.6)	94 (15.8)	135 (32.8)
*P* value^c^	.70	.80	.98
**Heard nutrition information from given source during the past 12 months^d^**			
Television			
A lot/some	969 (95.0)	212 (20.1)	266 (29.9)
A little/not at all	580 (92.9)	129 (18.9)	155 (25.2)
*P* value^c^	.28	.60	.14
Newspapers			
A lot/some	741 (94.8)	165 (21.1)	204 (30.6)
A little/not at all	704 (94.4)	149 (17.9)	188 (26.1)
*P* value^c^	.83	.20	.21
Magazines			
A lot/some	912 (95.7)	218 (21.8)	251 (28.5)
A little/not at all	530 (92.9)	97 (16.2)	140 (28.3)
*P* value^c^	.20	.06	.97
Internet			
A lot/some	417 (98.0)	119 (24.5)	98 (22.4)
A little/not at all	642 (95.4)	130 (15.9)	167 (24.9)
*P* value^c^	.13	.009	.47
Health care professionals			
A lot/some	581 (93.2)	144 (23.8)	170 (32.3)
A little/not at all	996 (94.9)	204 (17.0)	257 (25.6)
*P* value^c^	.33	.01	.02
**Age, y**			
18-34	874 (83.9)	153 (12.2)	233 (29.4)
35-49	1,322 (87.7)	306 (19.7)	359 (33.2)
50-64	1,381 (90.6)	331 (19.8)	404 (34.8)
65-74	717 (89.4)	152 (19.4)	225 (41.0)
≥75	539 (76.4)	73 (11.8)	184 (46.4)
*P* value^c^	<.001	<.001	<.001
**Body mass index^e^ (kg/m^2^)**			
Mean body mass index	27.2	29.1	27.4
*P* value^c^	<.001	.01	<.001

Abbreviation: LCDs, low-carbohydrate diets.

a Sample sizes vary by item because of missing data; responses of "don't know" and "refused" were coded as missing.

b Includes all participants who responded that they had tried an LCD during the past 12 months. We classified respondents who reported not being aware of LCDs (n = 584) as not having tried LCDs.

c *P* values derived from χ^2^ test of independence.

d Inclusion of nutrition-related behavior and information-seeking questions in the multivariate model substantially reduced the sample sizes because only half of the total sample was randomized to receive these questions.

e Body mass index calculated as [weight (lb)/(height [in])^2^] x 703; respondents self-reported weight in pounds and height in feet and inches.

**Table 2:** Correlates of Awareness, Use, and Perceived Healthfulness of Low-Carbohydrate Diets, Health Information National Trends Survey (HINTS), United States, 2005

Respondent Characteristic	Model A	Model B	Model C

	Aware of LCDs (n = 1,658), OR (95% CI)	Use LCDs^a^ (n = 1,042), OR (95% CI)	Believe LCDs Are Healthy (n = 935), OR (95% CI)
**Sex**			
Male	Ref	Ref	Ref
Female	1.47 (0.87-2.51)	1.67 (1.09-2.55)	0.71 (0.45-1.10)
*P* value	.15	.02	.12
**Age**	NA	1.00 (1.00-1.00)	1.00 (0.98-1.03)
*P* value	NA	.99	.84
**Education level**			
<High school	Ref	Ref	Ref
High school graduate	3.48 (1.64-7.37)	1.25 (0.27-5.92)	0.23 (0.09-0.56)
College graduate	10.10 (3.82-26.75)	1.64 (0.32-8.53)	0.17 (0.06-0.45)
*P* value	<.001	.34	.003
**Race/ethnicity**			
Non-Hispanic white	Ref	Ref	Ref
Non-Hispanic black	0.16 (0.08-0.34)	0.86 (0.34-2.18)	1.88 (0.79-4.47)
Hispanic	0.24 (0.13-0.47)	1.70 (0.48-6.02)	1.32 (0.53-3.29)
Other	0.38 (0.11-1.31)	0.64 (0.18-2.20)	2.17 (0.79-5.97)
*P* value	<.001	.69	.15
**Annual household income^b^**	1.00 (0.99-1.01)	1.00 (0.99-1.00)	0.99 (0.99-1.00)
*P* value	.69	.45	.06
**Body mass index^c^**	1.09 (1.02-1.17)	1.06 (1.03-1.10)	0.99 (0.95-1.03)
*P* value	.01	<.001	.70
**Change behavior according to new nutrition recommendations^d^**			
No	Ref	NA	Ref
Yes	0.62 (0.33-1.15)	NA	3.04 (1.88-4.91)
*P* value	.13	NA	<.001
**Information from Internet^d^**			
A lot/some	NA	Ref	NA
Little/not at all	NA	1.00 (0.66-1.51)	NA
*P* value	NA	.02	NA
**Information from health care professional^d^**			
A lot/some	NA	Ref	Ref
Little/not at all	NA	0.61 (0.40-0.93)	0.82 (0.56-1.19)
*P* value	NA	.99	.29

Abbreviations: LCDs, low-carbohydrate diets; OR, odds ratio; CI, confidence interval; Ref, referent; NA, not applicable.

a Includes all participants who responded that they had tried an LCD in the last 12 months.

b The 10-level categorical variable for income was treated as continuous in this model.

c Body mass index calculated as [weight (lb)/(height [in])^2^] x 703; respondents self-reported weight in pounds and height in feet and inches.

d Inclusion of nutrition-related behavior and information-seeking questions in the multivariate model substantially reduced the sample sizes because only half of the total sample was randomized to receive these questions. Information-seeking questions about the Internet and health care professionals referred to activities performed during the past 12 months.
